# New Insights into Gastrointestinal Involvement in Late-Onset Pompe Disease: Lessons Learned from Bench and Bedside

**DOI:** 10.3390/jcm10153395

**Published:** 2021-07-30

**Authors:** Aditi Korlimarla, Jeong-A Lim, Paul McIntosh, Kanecia Zimmerman, Baodong D. Sun, Priya S. Kishnani

**Affiliations:** 1 Division of Medical Genetics, Department of Pediatrics, Duke University Medical Center, Durham, NC 27710, USA; jeonga.lim@duke.edu (J.-A.L.); baodong.sun@duke.edu (B.D.S.); 2 Department of Neurology, Duke University Medical Center, Durham, NC 27710, USA; paul_mcintosh@duke.edu; 3 Duke Clinical Research Institute, Durham, NC 27710, USA; kanecia.zimmerman@duke.edu

**Keywords:** late-onset Pompe disease, gastrointestinal, smooth muscles, PROMIS–GI symptom scales, GAAKO mice, glycogen storage disorder, translational research, patient-reported outcomes measures

## Abstract

Background: There are new emerging phenotypes in Pompe disease, and studies on smooth muscle pathology are limited. Gastrointestinal (GI) manifestations are poorly understood and underreported in Pompe disease. Methods: To understand the extent and the effects of enzyme replacement therapy (ERT; alglucosidase alfa) in Pompe disease, we studied the histopathology (entire GI tract) in Pompe mice (GAAKO 6^neo^/6^neo^). To determine the disease burden in patients with late-onset Pompe disease (LOPD), we used Patient-Reported Outcomes Measurements Information System (PROMIS)-GI symptom scales and a GI-focused medical history. Results: Pompe mice showed early, extensive, and progressive glycogen accumulation throughout the GI tract. Long-term ERT (6 months) was more effective to clear the glycogen accumulation than short-term ERT (5 weeks). GI manifestations were highly prevalent and severe, presented early in life, and were not fully amenable to ERT in patients with LOPD (*n* = 58; age range: 18–79 years, median age: 51.55 years; 35 females; 53 on ERT). Conclusion: GI manifestations cause a significant disease burden on adults with LOPD, and should be evaluated during routine clinical visits, using quantitative tools (PROMIS-GI measures). The study also highlights the need for next generation therapies for Pompe disease that target the smooth muscles.

## 1. Introduction

Pompe disease (glycogen storage disease type II, OMIM ID: 232300) is an autosomal recessive disorder caused by deficiency of the enzyme acid α-glucosidase (GAA) [1]. This deficiency leads to an abnormal accumulation of glycogen in the cardiac, skeletal and smooth muscles, and the nervous system. Pompe disease is broadly classified as infantile-onset (IPD) or late-onset Pompe disease (LOPD) [2]. Patients with IPD have little or no GAA enzyme activity, resulting in cardiomyopathy in the first year of life, and if untreated, die from cardiorespiratory complications before two years of age [2]. Patients with LOPD have residual GAA activity, and present with a slowly progressive myopathy and respiratory failure, with symptom onset ranging from the first year of life to the sixth decade [2,3]. Enzyme replacement therapy (ERT; alglucosidase alfa) is the standard of care for IPD and LOPD. Prior to its advent in 2006, LOPD was considered a proximal limb girdle muscle dystrophy with pulmonary involvement [4]. Over time, there has been a growing evidence of smooth muscle involvement in individuals with Pompe disease with reports of life-threatening basilar artery and ascending aorta aneurysms, difficulties in swallowing and speech, and the involvement of eyes, genitourinary tract, and gastrointestinal (GI) tract [3,5,6,7,8,9].

GI manifestations are poorly understood, often underreported, or misdiagnosed as a separate entity [10,11,12]. GI manifestations in LOPD include abdominal pain, feeding and swallowing difficulties, gastroesophageal reflux, postprandial bloating, early satiety, abdominal discomfort, chronic diarrhea, constipation, poor weight gain, and decreased gag reflex [7,13,14,15]. Patients with LOPD were found to have significantly more stool urgency, incontinence, and diarrhea, when compared to age- and gender-matched controls [7,13,16,17]. There are a few case reports and small case series describing improvement in GI symptoms with ERT therapy [11,14,15]. However, objective evidence of glycogen clearance within the GI tract is lacking. This could be attributable to the inefficient delivery of ERT to the target tissues (skeletal and smooth muscles). Therefore, many patients on long-term ERT still encounter a multitude of clinical symptoms, such as skeletal muscle weakness, respiratory failure, sleep disturbances, gastro-intestinal (GI), and genitourinary problems.

Autopsy data from patients with Pompe disease show a mild to moderate accumulation of glycogen in the tongue (skeletal muscles) and proximal third of esophagus (striated muscles) contributing to dysphagia, and in the smooth muscles of the distal esophagus and small intestines causing gastrointestinal symptoms [18,19,20]. Severe fibrosis, dilatation, increased vacuolization of myocytes, and autophagic buildup were noted in the esophagus in two adult patients with LOPD [18,21]. Although three available Pompe disease knockout (GAAKO) mice are extensively used in preclinical studies, the entire GI tract and its response to ERT have not been studied. Data from two of three mouse models show extensive glycogen accumulation in the stomach, small intestine, and colon (including the nervous supply or plexus) in a 15-month-old Δ13/Δ13 model, generated by the targeted disruption of exon 13; and glycogen accumulation in the esophagus in a 6-month-old 6^neo^/6^neo^ model, generated by the targeted disruption of exon 6 [6,22,23,24].

Therefore, there are unmet needs to systematically understand the spectrum of GI involvement, the histopathology of the entire GI tract, and the impact of the available treatment (ERT) on Pompe disease. The aims of this study were (a) to better understand the wide range of GI symptoms, including their frequency and severity, as well as the disease burden in adult patients with LOPD using patient-reported outcomes, and (b) to assess the distribution of glycogen accumulation within the entire GI tract, and study the effects of ERT using the 6^neo^/6^neo^ GAAKO mouse model.

## 2. Materials and Methods

The study design included the use of patient-reported outcome measures to understand the prevalence and severity of GI disorders (Section 2.1), and the use of a mouse model to understand the histopathology of the entire GI tract (Section 2.2).

### 2.1. Participants

All participants were enrolled in a long-term follow up study of Pompe disease (Pro00010830) at the Duke University Medical Center. The study protocol was approved by the Duke University Institutional Review Board (Pro00010830). Eligible participants were adults (ages ≥ 18 years) with a confirmed diagnosis of LOPD (*n* = 58), who were evaluated at Duke between April 2017 and July 2018. Written informed consent was obtained from each participant prior to all assessments.

The GI health of all the participants was prospectively evaluated during their routine clinical visits to Duke University. For participants who were evaluated more than once during the study period, their baseline data were used in the cross-sectional analysis, and the follow-up data were used in the longitudinal analysis of the study. Participants completed a GI questionnaire (Patient-Reported Outcomes Measurements Information System—Gastrointestinal (PROMIS-GI) symptom scales) and/or a GI-focused medical history was obtained by one medical geneticist (P.S.K.) during the same clinical visit, depending on the time available during clinic.

#### 2.1.1. PROMIS-GI Symptom Scales

The PROMIS-GI symptom scales v1.0 are validated, person-centered questionnaires designed to assess patient-reported quality of life due to GI dysfunction, available on the HealthMeasures website (http://www.healthmeasures.net/explore-measurement-systems/promis, accessed on 15 February 2021), which is funded by the National Institutes of Health (NIH) [25,26,27,28]. There are eight PROMIS-GI scales available as ‘fixed-length, short forms’ for adult participants, with a designated unique name and number/letter—Gastrointestinal Disrupted Swallowing 7a, Gastroesophageal Reflux 13a, Gastrointestinal Gas and Bloating 13a, Gastrointestinal Belly Pain 5a, Gastrointestinal nausea and vomiting 4a, Gastrointestinal Bowel Incontinence 4a, Gastrointestinal Diarrhea 6a, and Gastrointestinal Constipation 9a. The current study used all eight available GI scales. These eight GI scales comprise a total of 54 items. Each item has a five-point categorical response (for example: 1 = never, 2 = rarely, 3 = sometimes, 4 = often, and 5 = always to evaluate severity, and frequency scales to evaluate frequency). Based on these categorical responses, a free, automated scoring system (HealthMeasures Scoring Service) and a manual scoring guide (https://www.healthmeasures.net/images/PROMIS/manuals/PROMIS_Gastrointestinal_Symptoms_Scoring_Manual.pdf; last accessed on 3 June 2020) were used to calculate statistical scores (raw and T-scores) [27,28]. In addition, HealthMeasures provides two reference populations to evaluate the PROMIS-GI measures—General population (GP; *n* = 1177 persons from the 2010 United States (US) census, who reported at least 1 GI symptom) and GI clinical sample (*n* = 865 patients with GI conditions) [27,28].

Raw scores: Raw scores were used to measure the prevalence of GI symptoms in this patient population. Based on the five-point categorical response, each item was rated 1 to 5, where 1 meant that the GI symptom was absent and a higher score (2–5) meant that a symptom was present with increasing severity and/or frequency. The item scores on each GI symptom scale were then summed to obtain a raw score. Therefore, each patient received one raw score for each GI scale. The scoring manual was then used to obtain a cut-off raw score for each GI scale, which indicated that a patient was symptom-free on a certain GI scale if they were at the cut-off value, or had GI problems if they scored above the cut-off value (depending on how many items were answered or skipped). Based on this, a ‘yes’/‘no’ analysis was conducted for each GI scale. If a patient reported having a problem within one GI scale, it was considered a ‘yes’ response; if the patient was symptom-free, a ‘no’ response was recorded. For instance, PROMIS-GI Bowel Incontinence scale includes four items. If a patient responds to all four items, and has no problems related to bowel incontinence, the summed raw score for GI Bowel Incontinence scale would be 4 (the cut-off value). Therefore, a raw score of 4 would be a ‘no’ response to the GI Bowel Incontinence domain. Any score over 4 would indicate that there was some problem in the domain, and therefore, reflective of a ‘yes’ response.

T-scores: T-scores were used to understand the severity and prevalence of the GI symptoms. The mean T-score for the control group (US GP) was 50 with one standard deviation (SD) of 10 [27,28]. The T-scores from patients with LOPD were compared to T-scores of the reference populations (US general population and GI clinical sample) [25,26,27,28]. T-scores ranging between 55 and 59 were considered mildly symptomatic, 60–69 were moderate, and over 70 were severe, based on the available scoring guides.

To understand the impact of the GI symptoms on patients over time, baseline (first clinic visit) and follow-up (subsequent visits) T-scores were compared. The differences between the baseline and follow-up T-scores and minimally important differences (MIDs) were computed based on PROMIS databases [27]. These MIDs are estimates for the magnitude of change that corresponds to meaningful changes for patients with a specific GI symptom [29]. The estimated reference values for MIDs for each GI scale are provided on the HealthMeasures website. A change of 5–6 points (T-score) between two time points (for gas and bloating, belly pain, diarrhea, and constipation scales) would be indicative of significant clinical change in the specific GI symptom. For instance, a participant with a baseline T-score of 60 on belly pain and a follow-up T-score of ≥66 for the same scale (belly pain) would indicate clinically significant worsening. However, if the participant had a follow up T-score of ≤54, it would indicate improvement for belly pain. These are available on the HealthMeasures website.

#### 2.1.2. GI-Focused Medical History

The GI-focused medical history was pre-designed for the current prospective study to assess the GI health of adult patients with Pompe disease. It included 16 questions, which PSK asked the participants during their clinic visits (Supplementary Table S1). The questions provided details of GI symptoms (if present), their associations with meals, diurnal variations, medications taken for GI discomfort, whether the onset of GI symptoms was before or after the diagnosis of LOPD, any changes in GI symptoms in ERT-treated patients, and whether the participants considered their GI symptoms to be one of the top three reasons to cause a reduced quality of life. It also included history of tongue weakness, chewing problems, and temporomandibular joint issues made by other medical professionals.

Medical records were reviewed to include ERT doses, age at ERT initiation, and the duration of treatment with ERT, and most recent values of creatine phosphokinase (CPK), 6-min walk test distance (6-MWT), FVC % predicted (upright), FVC % predicted (supine), and urinary hex4 (a breakdown product of glycogen, which is a biomarker of disease progression), which were available during the study period.

#### 2.1.3. Statistical Analyses

Descriptive statistics were used to summarize the distribution of categorical variables using counts (percentages) and medians (25th and 75th percentiles) to analyze the GI-focused medical history and the responses on individual items within the PROMIS-GI questionnaire. Where appropriate, a one-sample test was used to compare T-scores to the reference T-score = 50. Pearson’s chi-squared test was used to compare GI symptoms in patients who were on ERT to those who were not on ERT during the study period. A *p*-value of ≤0.05 was considered statistically significant for the *t*-test and chi-squared analyses. For all other analyses, we used Bonferroni correction for comparisons to identify a significant *p*-value of <0.006.

To understand the role of ERT on GI symptoms in LOPD, participants who completed the PROMIS-GI questionnaires were divided into two groups. Group I consisted of patients treated with ERT < 6 months + untreated. Patients with less than 6 months ERT were included in this group to account for the time it takes for ERT to show clinical benefits. Group II included patients who were treated ≥ 6 months with ERT. Using the two-sample Wilcoxon rank-sum (Mann–Whitney) test, we compared the T-scores/raw scores on each of the eight GI symptom scales between the two groups. The Wilcoxon rank-sum (Mann–Whitney) test was also used to explore statistical relationships between each of the eight GI T-scores/raw scores (for each group) with patient’s age, sex, age at diagnosis, age at ERT start, and most recent values of CPK, 6-MWT, FVC % predicted (upright), FVC % predicted (supine), and urinary hex4.

### 2.2. GAAKO Mouse Model (6^neo^/6^neo^)

Animal care and experiments were conducted in accordance with Duke University Institutional Animal Care and Use Committee-approved guidelines. To study the extent of GI pathology, 3-month-old male GAAKO (6^neo^/6^neo^) mice were used. Age- and gender- matched wild type (WT) mice were used as a control. To understand the short-term effects of ERT on GI smooth muscles, a 3-month-old GAAKO mice received 20 mg/kg ERT (hGAA, alglucosidase alfa, Myozyme) through the tail vein every week for 5 weeks. Phosphate-buffered saline (PBS)-injected GAAKO mice were used as control (ERT-naïve or placebo group). To understand the long-term effects of ERT on GI smooth muscles, a 2-month-old GAAKO mouse model received 20 mg/kg ERT through the tail vein, biweekly for 6 months.

#### Histopathology

The following anatomical regions of the GI system were analyzed in the mice: tongue, upper 1/3rd of the esophagus, lower 1/3rd of the esophagus, stomach, gastro-esophageal (GE) junction, duodenum, small intestine (jejunum, ileum, cecum, and ileo-cecal junction), colon, and rectum.

GI tissues were fixed in 10% neutral-buffered formalin (NBF) for 48 h. After primary immersion fixation, the samples were post-fixed with 1% periodic acid in 10% NBF for 48 h at 4 °C. The samples were then washed with PBS, dehydrated with ascending grades of alcohol, cleared with xylene, and infiltrated with paraffin. Sections of paraffin-embedded tissues were stained using a Periodic acid-Schiff (PAS) stain as described [30]. Briefly, the sectioned slides were deparaffinized, re-hydrated, and oxidized with freshly made 0.5% Periodic acid for 5 min. The slides were then stained with Schiff reagent for 15 min and washed with tap water for 10 min. The tissues were counterstained with hematoxylin, dehydrated, and mounted. Paraffin-embedded sections were also processed and stained using Masson’s trichrome staining kit (Sigma-Aldrich Co., St. Louis, MO, USA) following the manufacturer’s protocol. The images were taken on a BZX710 microscope (Keyence America, Itasca, IL, USA).

The PAS was used to detect glycogen content within the cells of the tissues. The cells with an accumulation of glycogen stain dark pink/purple and the cell nuclei stain blue. The Masson’s trichrome staining was used to explore the presence and extent of tissue fibrosis, which stains blue. Muscle fibers and cytoplasm stain red, and the cell nuclei stain dark brown.

## 3. Results

### 3.1. Participants

Patient demographics are shown in Table 1. Whenever possible, each patient completed the PROMIS-GI questionnaires, and the clinician could obtain the GI-focused medical history during the routine clinical visits. However, due to time constraints, certain patients either only completed the PROMIS-GI questionnaire or the GI-focused history was obtained. Data analysis based on the PROMIS-GI scales are shown in Section 3.1.1 (prevalence and severity using raw scores) and Section 3.1.2 (comparisons to reference population and longitudinal analysis mainly using T-scores).

#### 3.1.1. PROMIS-GI Symptom Scales

##### Raw Scores

**Figure 1:**
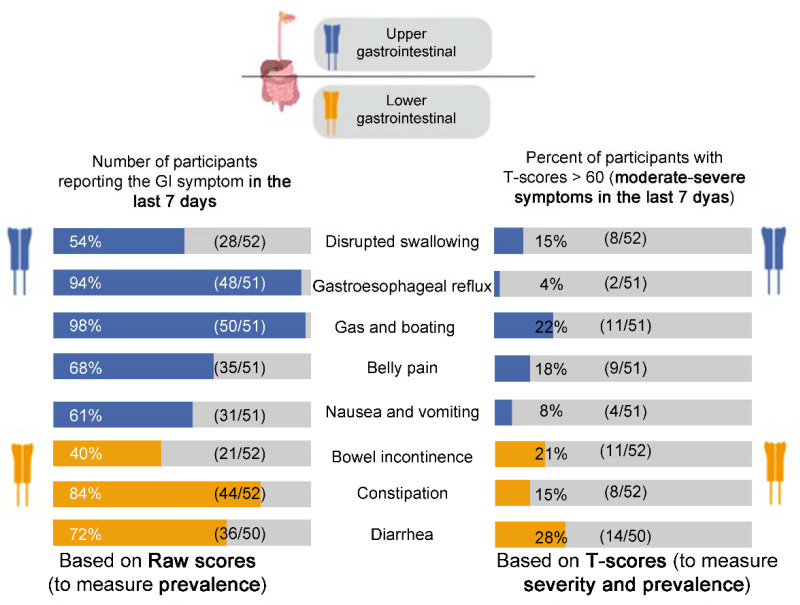
The prevalence and severity of gastrointestinal symptoms in adult patients with late-onset Pompe disease using PROMIS-GI symptom scales (This figure was created using BioRender.com; accessed on 5 January 2021).

Using the ‘yes/no’ analysis on the raw scores, the prevalence of each GI symptom in adult patients with Pompe disease were calculated and compared to the reference populations. Details are shown in Figure 1 and Table 2.

##### T-Scores

Severity was computed for each GI scale. Figure 1 demonstrates the prevalence of patients with moderate to severe grades (T-scores > 60 or ≥1 SD compared to the reference population) for each GI scale. The prevalence of moderate-severe GI symptoms ranged from 4 to 28% (Figure 1). The mean T-scores (SD) for each GI scale at baseline were calculated and compared to the reference populations (Table 2). The mean T-scores ranged from 46.34 (belly pain) to 54.57 (gas and bloating) in the cross-sectional analyses. Though these values were not significantly different from the reference populations, the longitudinal analyses (MID) yielded meaningful, clinically significant results in a subset of patients (Table 2). These calculated MIDs indicated a clinically significant change in the GI symptoms over the study period, where some patients showed improvement, worsening, or no change in their symptoms.

#### 3.1.2. GI-Focused Medical History

The 16 clinical questions *(*Supplementary Table S1*)* revealed details about the GI-symptoms, aggravating or relieving factors (diurnal variations, diet, and medications), and overall subjective perception of quality of life due to GI problems in individuals with LOPD. In the current study, 29/38 (76%) patients reported at least one GI problem, and 23 of those 29 patients (82%) reported that there were no changes in their GI symptoms after initiation of ERT. Five patients reported some changes in their GI symptoms; two felt better with additional GI medications (CoQ10 and probiotics for diarrhea, laxatives for constipation), one felt that the symptoms were reduced after an increase in the dose of ERT (from 20 to 40 mg/kg), and two had worsening symptoms with increased diarrhea and GE reflux while on ERT.

Over half of the patients with LOPD, 21/38 (55%), were on additional medications to manage their GI symptoms. The medications included antacids, anti-diarrheal, anti-spasmodic, CoQ10, probiotics (which improved diarrhea in one patient), tincture of opium, and bulking agents, such as psyllium, methylcellulose, and polyethylene glycol, stool softeners such as docusate sodium, and linactolide to treat constipation. Eleven patients reported that the GI symptoms worsened with meals.

When asked ‘Did your GI symptoms start bothering you before or after your diagnosis of Pompe disease?’, 16/38 (42%) patients indicated before and 14/38 (37%) indicated after the LOPD diagnosis. The rest of the patients (8/38; 21%) either could not recollect the onset of their GI manifestations or did not have a GI problem. Of the 16 patients who reported that GI symptoms presented before the LOPD diagnosis, 6 recollected that they were in their 20s at onset, one was in their 40s, and three patients had the GI symptoms since their childhood years; all 6 of these patients reported that they had no improvements in GI symptoms with ERT. Though patients could not recall the exact age at onset of GI symptoms, their responses indicated that age at onset of GI symptoms ranged from childhood to the seventies in the cohort. In addition, when asked ‘Do you consider your GI symptoms to be one of your top three symptoms that affects your quality of life?’, 15/38 (40%) patients replied ‘yes.’

Six patients reported that they had worsening GI symptoms within 48 h of ERT infusion. All six patients reported that these were isolated (one-time) episodes, and only four of them were able to recollect the details (one patient had vomiting, two had diarrhea, and another had an upset stomach). None of these episodes reoccurred in any of the six patients.

#### 3.1.3. Statistical Analyses

Gas and bloating was the only GI symptom scale (of the eight) to yield a significant *p*-value (0.0014) when the T-scores were compared to the US general population. Using Bonferroni correction for comparisons (significant *p*-value of <0.006) between the untreated (*n* = 6) and the treated group (*n* = 46), there were no statistically significant differences on the eight GI scales. The only exception to this was the relationship between the GI symptom scale of swallowing (higher raw scores) and FVC % predicted (low values), which yielded a *p*-value of 0.0036score. Other important relationships, albeit not statistically significant, are listed in Supplementary Table S2.

### 3.2. GAAKO Mouse Model (6^neo^/6^neo^)

#### Histopathology

**Figure 2:**
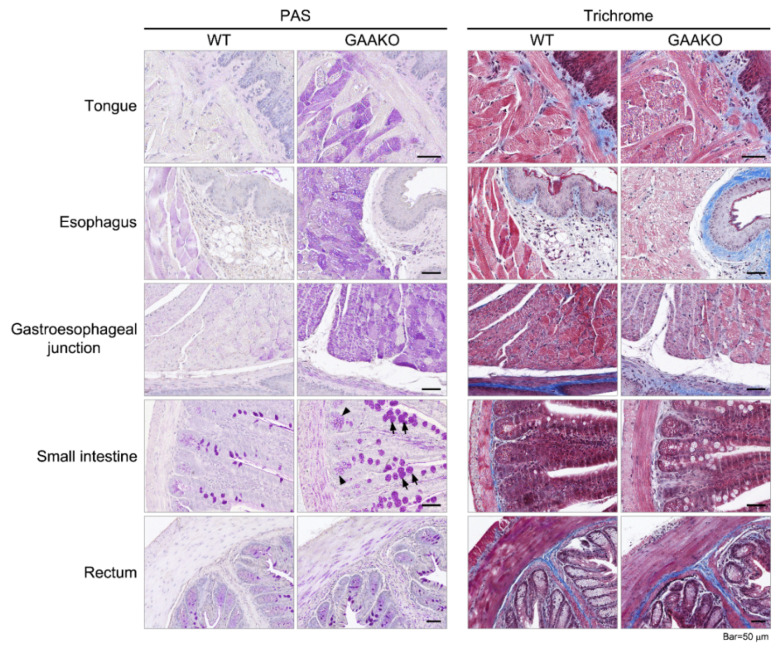
Periodic acid-Schiff (PAS) and Trichrome staining of the gastrointestinal tissues in Pompe mice (GAAKO), using wild type (WT) mice as controls. Extensive glycogen accumulation in the GAAKO mice, when compared to the WT mice (purple-stained skeletal muscles of the tongue and esophagus, and the smooth muscles in the esophagus, gastroesophageal junction, small intestine, and rectum). The intestinal villi (small intestine) showing hypertrophic and mild hyperplastic goblet cells (arrows) and intestinal glands (arrowheads) can be seen.

The PAS staining showed that there was glycogen accumulation through the entire length of the GI tract (from the tongue to the rectum) in the GAAKO mouse model (Figure 2). Glycogen accumulation was seen in the smooth muscles of the esophagus, gastroesophageal junction, small intestine, rectum, duodenum, cecum, and colon. The smooth muscle layers of the submucosa and muscularis externa were the most affected (Figure 2).

**Figure 3:**
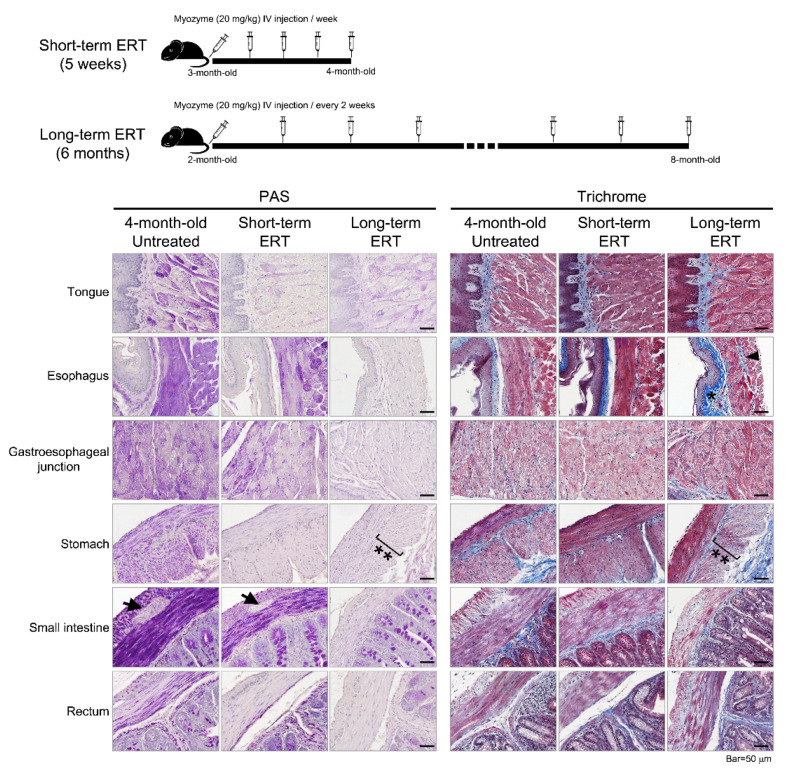
Short-term and long-term effects of ERT (alglucosidase alfa) on Pompe mice (GAAKO). ** Distorted muscularis externa layer in the stomach. * Fibrosis was noted in the 8-month-old mice; however, this was also seen in the wild type mice. Aurbach’s plexus was seen in between the inner circular and outer longitudinal layers of the small intestine (arrows) and in the esophagus (arrowheads).

The WT mice did not have any glycogen accumulation. The glandular portions of the stomach and small intestine showed disintegrated ganglion cell structures (Aurbach’s plexus) in the GAAKO mice (visualized in Figure 3).

The small intestine of the GAAKO mouse model showed hypertrophic goblet cells with hyperplasia. Duodenal sections showed hypertrophic villi in addition to the glycogen accumulation. The cecum of the GAAKO mouse model showed a mild disruption of the brush border of the epithelial lining and vacuolated nuclei of the enterocytes. The rectal section of the GAAKO mice showed some neutrophilic accumulation in the lamina propria. There was no significant sign of fibrosis in the GI tract in the GAAKO mice when compared to WT.

The effects of ERT (alglucosidase alfa) on the GI tissue in GAAKO mice were also evaluated. The short-term (5 weeks) treated mice showed clearance of the glycogen accumulation in tongue, stomach, and rectum (Figure 3). However, the smooth muscle in the esophagus, gastroesophageal junction, and small intestine still showed glycogen accumulation. The glycogen accumulation in Aurbach’s plexus in the small intestine was cleared by the short-term ERT. There was no sign of fibrosis on trichrome staining in the GAAKO mice.

With long-term ERT (6 months), there was a reduction of glycogen accumulation in the entire GI tract (Figure 3). Fibrosis was seen in the old mice in the esophagus, smooth muscles of the GEJ, and submucosal region of the stomach. Fibrosis was also observed in the WT mice. The inner circular muscular layer of the stomach looked distorted, and had less muscle density (hypotrophic). Though structurally organized, the Aurbach’s plexus in the esophagus had some mild fibrosis.

## 4. Discussion

LOPD is a chronic, multi-systemic disorder, with a substantial burden on health and quality of life of the patients and their caregivers [31,32]. A large number of patients with LOPD complain about GI symptoms in the clinics [7,9,11,13,14,32]. However, there still exists a knowledge gap about the impact of the GI system on LOPD, the extent of gut involvement, severity, prevalence, and treatment response to standard dose of ERT with alglucosidase alfa. There can be several factors that may influence the presence and severity of GI symptoms in LOPD, such as genotype-phenotype correlations, patient’s age, age at ERT start, and overall disease burden, which can be evaluated through pulmonary function testing or muscle weakness. Patients with LOPD exhibit variable rates of progression of myopathy and pulmonary compromise, and early ERT initiation has been shown to have better outcomes compared to the untreated patients [33]. Similarly, with variable presentations of GI manifestations in LOPD, more research is needed to understand if early treatment would impact the outcomes. With an aim to bridge these knowledge gaps, the current study used Pompe mice and patient-reported outcomes measures (PROMIS-GI scales and GI-focused history).

The PROMIS-GI scales provide patient-reported information about the physical, mental, and social health related to a spectrum of GI manifestations, and therefore, by definition, provided preliminary data on quality of life [27,28,34]. The raw and T-scores from these PROMIS-GI scales indicated that the GI manifestations in LOPD were highly prevalent and severe (Figure 1, Table 2). The GI-focused medical history showed that 40% patients with LOPD (15/38) considered their GI symptoms to be one of the top three reasons affecting quality of life, and that 55% patients (21/38) were on additional medications to treat their GI symptoms, despite being on ERT. Overall, the current study showed that GI manifestations remarkably reduced quality of life in adults with LOPD.

Interestingly, the study showed that GI manifestations preceded the LOPD diagnosis in 42% (16/38), and the age of onset of GI symptoms ranged from childhood to their 40s in 6/16 patients. The GI symptoms may be an early manifestation of the disease, and their presence could be used as an adjunct to monitor disease progression, and to consider a diagnosis of Pompe disease. More than ever, monitoring disease manifestations and progression is important in LOPD with its inclusion in newborn screening programs, and with improved diagnostic criteria [35]. The use of the PROMIS-GI measures in routine follow-ups could provide useful information about clinical progression of the disease and the effectiveness of emerging therapies [36]. In addition, as GI symptoms are often under-reported by patients, awareness and focused history taking can alert clinicians to refer patients to GI specialists, when required.

Using the PROMIS-GI scales, the current study used two reference populations—the US general population and a GI sample (Figure 1, Table 2). In the US general population, there was notably a high population prevalence of GI symptoms [28,29]. The current study showed that patients with LOPD had a much higher prevalence in comparison to both reference populations—gas and bloating (98%), gastroesophageal reflux (94%), constipation (84%), diarrhea (72%), belly pain (68%), nausea and vomiting (61%), disrupted swallowing (54%), and bowel incontinence (40%) (Figure 1). This was based on raw scores. Based on T-scores, there was no significant difference in the prevalence when compared to the reference populations (Table 2). Therefore, at the population level, there was no significant difference in prevalence of GI symptoms comparing the LOPD and two reference groups. However, at the individual level (each patient with LOPD, and each GI scale as a unique symptom), the prevalence of a GI symptom requires close attention (Figure 1). Therefore, to better understand the impact and severity of each GI symptom on patients with LOPD, it is important to evaluate each patient on a case-by-case basis (individual) as well as a group (population/cohort). For instance, gastroesophageal reflux was highly prevalent (94%) in the LOPD group; of these, 4% of the patients had moderate to severe symptoms. On the other hand, while diarrhea was prevalent in 72% patients, 28% of those patients suffered from moderate to severe diarrhea. This shows that diarrhea as a presenting symptom may require early and prompt medical attention to avoid progression to severe diarrhea. Therefore, using these quantitative screening tools (PROMIS-GI and GI-focused history), all patients with LOPD should be routinely screened for GI problems during the first clinical visit and in subsequent follow-ups.

Longitudinal analyses in the current study (*n* = 19) showed that over time, a majority of patients had no meaningful changes (computed MIDs) in the GI scales. When the MID was identified, worsening or improvement was reported by patients in roughly equal numbers (Table 2). However, when ‘worsening’ and ‘no change’ MIDs were taken together, it indicated that ERT and the additional GI medications (over the counter) may be inefficient to treat the GI symptoms in patients with LOPD. This was further substantiated with the data from the GI-focused medical review, which suggested that 21/29 patients with at least 1 GI symptom (82%) reported no changes in their GI symptoms over time, even after the initiation of ERT (median duration of ERT = 5.5 years; range = 2 months–13 years).

Six patients reported that they had worsening GI symptoms within 48 h of ERT infusion, as per the GI-focused medical review. To understand if this could be related to GI-related adverse reactions from the ERT infusions, we checked the package insert of ERT (Lumizyme, US Food and Drug Administration). The Lumizyme package insert of ERT defines ‘infusion reactions’ as adverse reactions that occurred during or within 2 h of ERT infusion, and ‘delayed-onset infusion reactions’ occurred within 48 h [37]. As per the findings from their controlled study, GI-related infusion reactions in the LOPD group (*n* = 60) included constipation (*n* = 6), dyspepsia (*n* = 5), and vomiting (*n* = 13). There were no GI-related ‘delayed onset’ infusion reactions. Thus, combining the clinical data from the controlled study in the Lumizyme package insert and the current study, certain GI symptoms may be temporally associated with ERT infusions. However, this temporal association can be made only for isolated adverse events such as the ones that were reported by the six patients in the current study.

Findings from patients with other inborn errors of metabolism, such as Fabry disease and Gaucher disease, suggest that 6–7 months of treatment with agalsidase beta and ceredase ERT, respectively, led to a marked improvement in the GI symptoms [38,39]. When compared to Fabry and Gaucher-type I diseases, patients with LOPD seem to be less responsive to ERT. The high prevalence of GI symptoms from treated patients (*n* = 46) and longitudinal data from 19 patients showed that despite being on ERT, GI involvement causes a huge disease burden on adults with LOPD. All these data are contrary to the data from previous small case series, which reported that patients with LOPD (*n* = 9) had a substantial improvement in their GI symptoms following the initiation of ERT for 3–12 months [7,11,14,15]. The current study highlights that the current doses of ERT (20 mg/kg biweekly) may be insufficient to target GI symptoms effectively, as shown by no clinical change or worsening in several patients. This response to ERT could be due to a number of factors, such as low density of mannose-6-phosphate receptors in skeletal and smooth muscles as compared to cardiac muscle [40,41], using lower than recommended dose of ERT, impaired autophagy [42,43], defective mitophagy leading to abnormalities in the cellular energy metabolism [44,45], an acidic shift in the cellular pH after lysosomal rupture [44,45], and reduced uptake due to scar tissue (or residual fibrosis) in the muscles. There is also a variable response to treatment due to muscle fiber type, angiotensin-converting enzyme insertion/deletion polymorphism, and polymorphisms in the ACTN3 gene (R577X). In addition, we analyzed important factors that may influence the severity and prevalence of GI symptoms in LOPD, namely, patient’s age, sex, age at diagnosis, age at ERT start, and most recent biomarkers of disease progression (CPK, 6-MWT, pulmonary function and urinary hex4). We did not identify any statistically significant relationships between these variables, potentially due to the small sample size (Supplementary Table S2).

Using the 6^neo^/6^neo^ GAAKO mouse model, the current study showed that the GI tract is involved in its entirety, from the tongue and esophagus to the rectum (Figure 2). Tissue injury in the mouse model included glycogen accumulation throughout the GI tract, with vacuolization, autophagy (shown in the stomach), and fibrosis. There was hypertrophy in the intestinal villi and hyperplasia of Goblet cells, which could be a sign of compensation for the loss of functioning. In addition, there was an involvement of Aurbach’s plexus (the nervous supply of the smooth muscles of the GI tissue) in the glandular portion of the stomach and small intestine. This further substantiates the need for alternate or adjuvant therapies with ERT which can target the smooth muscles and the nervous components. In the current study, short-term ERT (20 mg/kg per week for 5 weeks) could effectively clear the glycogen accumulation from the tongue, stomach, small intestine, and rectum (Figure 3). The short course of ERT also corrected the disintegrated cellular architecture in the Aurbach’s plexus of the stomach and small intestine. However, it was ineffective in clearing the glycogen accumulation in the other parts of the GI tract. This shows that ERT may be inefficient for clearing glycogen accumulation in GI smooth muscle when initiated at later stage of disease, even with a 2-fold increase in dosing (from standard dose). The long-term therapy with ERT (20 mg/kg biweekly for 6 months) was more effective in clearing the glycogen accumulation throughout the gut. The age at initiation of ERT was 2 months. This showed that during early stages of disease, if ERT is started and continued for a longer duration, ERT may be effective. These mouse data will help in translational studies in children with LOPD and IPD in the advent of the newborn screening era. More research is needed at this time to follow up older mice to understand the effectiveness of ERT on the gut when it is initiated at a later stage of the disease. This will provide a better understanding about age at ERT initiation in patients with LOPD. This is important because autopsy data from three adult patients with LOPD (ages 31 years, 53 years, and 62 years) showed mild–moderate glycogen accumulation in the esophagus and ileum and vacuolation and degeneration of tongue, upper and lower esophagus, ileum, and media of arterioles [18,20,21]. The skeletal portion of the upper esophagus showed glycogen accumulation, lipofuscin, neural lipid droplets, and autophagic debris as seen on electron microscopy from a 62-year-old female patient with LOPD [20]. These data suggest that there is a need for effective therapies that minimize the gut pathologies in LOPD, even if treatment is initiated when patients are in their 30s or 40s.

**Limitations**: The current study has its limitations. Though subjective (patient-reported) measures were used in the study, the inclusion of objective GI tests (such as endoscopy or manometry) was beyond the scope of the current study. Future studies with objective GI testing on patients with LOPD may substantiate the findings of cellular architecture (or histopathology) in the mouse model of the current study. In addition, nutrition and dietary habits cause significant changes in the GI health; however, these causative factors could not be evaluated at this time. The study raises a question about the effectiveness of ERT on patients with LOPD. However, due to the small sample size of patients with untreated group (*n* = 6), the study could not compare the untreated with the treated group for statistical analysis. Another limitation was that PROMIS database provide estimated MIDs only for only five GI symptom scales; the clinical significance and interpretation of changes in T-scores for three other symptom scales were still unknown during the study period. Moreover, since the PROMIS-GI scales are self-reports, missed or skipped items often cause biases in data analysis. However, the automated and the manual scoring guides, provided by HealthMeasures, are built in such a way that the biases caused by missed/skipped items in self-reports are eliminated [27,28,46]. The responses in each GI symptom scale are based on the patients experience in the previous seven days, rather than longer periods in time, to reduce recall biases. Keeping in mind the varied response options and literacy demands, the items are concise and simple worded [27,28,46]. Lastly, GAAKO mouse models may not be a true representative of LOPD (mimics the IPD phenotypes more). Moreover, the long-term therapy of 6 months in the mouse model is approximately 20–30 years of therapy in humans. The 20–30 years of ERT in humans may not be feasible in patients with LOPD. However, the 6^neo^/6^neo^ mouse model provided useful information about the cellular architecture in Pompe disease, and the impact of ERT on the GI tissue.

## 5. Conclusions

In conclusion, despite the limitations, the current study showed that about half the patients with LOPD had reduced quality of life due to GI symptoms, and these patients were on additional GI medications to treat these symptoms. However, most patients did not report any change or had worsening symptoms over time while being on ERT. Presumably, an earlier initiation of ERT may mitigate the development and progression of GI symptoms; however, this could not be concluded from the current study. Moreover, there is ineffective delivery of ERT to smooth muscles in the GI system [40,41,42,43,44], and the involvement of the neurological component of the gut (as seen by Aurbach plexus involvement in the current study). Recently, neurogenic dysfunction was shown in the urinary bladder of seven patients with LOPD, with probable causes of glycogen accumulation in the peripheral or central nervous system [16]. Due to a growing evidence of central nervous system involvement in Pompe disease, the neurological involvement in the GI system requires a closer evaluation [47]. In addition, the current study showed that the current recommended doses of ERT seem futile for maintaining GI health. Therefore, alternative therapies or second-generation drugs using gene therapy may be better ways to tackle the moderate to severe GI problems in patients with LOPD. In addition, since GI symptoms develop early in life, many times even before the diagnosis of LOPD, simple tools used in the current study (PROMIS-GI measures and GI-focused medical review) should be used to screen patients with clinical suspicion, and to observe progress of GI health in patients with LOPD as well as IOPD.

## Figures and Tables

**Table 1:** Patient demographics for the GI study in adult patients with LOPD.

**Study duration** **Total number of patients (*n*)** **(a)** **PROMIS-GI symptom scales** **(b)** **GI-focused medical history**	1 year, 3 months 58 *n* = 52 *n* = 38 (32/52 who also completed the PROMIS-GI + additional 6 patients who only had GI-focused medical history in their medical records)	
**Demographics**	Median age = 51.5 ± 15.5 years (age range: 18–79 years) 35 females, 23 males	
	**Patients on ERT (treated group)**	**Patients not on ERT (untreated group)**
	*n* = 53 Median age at start of ERT = 45.5 years (range: 11–77 years) Median ERT duration = 5.5 years (range: 2 months–13 years) *n* = 1/53 was included in the untreated group for statistical analysis since the duration of ERT was <6 months *	*n* = 4 ERT-naïve *n* = 1 discontinued ERT since 2–3 years, after being on ERT for 3 years (medical records indicated that patient had adverse effects of flushing, difficulty breathing, and GI symptoms of severe cramping, nausea, and diarrhea starting 2–3 days after each ERT infusion.
**For longitudinal analysis of PROMIS-GI scales**	*n* = 18 (1 baseline and 1 follow-up) *n* = 1 (1 baseline and 2 follow-ups)	

* to account for the time it takes for ERT to show clinical benefits. PROMIS-GI Patient-Reported Outcomes Measurements Information System—Gastrointestinal. ERT—Enzyme replacement therapy.

**Table 2:** GI problems in adult patients with late-onset Pompe disease using PROMIS-GI scales, when compared to the reference populations, and a measure of meaningful change in the T-scores of patients with Pompe disease on longitudinal analysis.

PROMIS-GI Symptom Domain		Prevalence (Using Raw Scores)			Prevalence/Severity [Using Mean T-Scores (SD)]			Minimally Important Differences (MIDs) in T-Scores (*n* = 19) ***			
		Study Population	Reference Population [29]		Study Population	Reference Population [28]					
		Patients with Pompe Disease *n* = 52	GP *n* = 1177	GI Clinical Sample *n* = 865	Patients with Pompe Disease	GP	GI Clinical Sample	Estimated Reference Values for MIDs [29]	*n* with Improvement	*n* with Worsening	*n* with No MID in T-Scores
Upper GI	disrupted swallowing	54%	5.8%	*u*	49.15 (9.60)	50 (10)	51 (10)	*u*	*N/A*		
	gastroesophageal reflux *	94%	16–30.9%	33%	46.76 (8.06)	50 (10)	51 (10)	+5 points for improvement, −1 point for worsening	4	6	9
	gas/bloating	98%	20.6%	*u*	54.57 (7.68)	50 (10)	57 (10)	±6 points	4	4	10
	belly pain	68%	24.8%	*u*	46.34 (12.06)	50 (10)	57 (11)	±6 points	4	5	9
	nausea/vomiting	61%	9.5–19%	24%	47.07 (7.35)	50 (10)	53 (10)	*u*	*N/A*		
Lower GI	constipation *	84%	19.7–47%	39% total	50.05 (8.54)	50 (10)	54 (10)	±5–6 points	2	4	12
	diarrhea	72%	6.6–20%	*u*	52.18 (10.38)	50 (10)	56 (11)	±5–6 points	3	2	13
	incontinence	40%	8.3%	*u*	48.67 (9.25)	50 (10)	53 (11)	*u*	*N/A*		
Other GI condition [28]	IBS * IBD * systemic sclerosis * others	*N/A*	11% 4% 1% 47%	40% 28% 18% 39% **	*N/A*			*N/A*			

GP—general population, GI—gastrointestinal, MID—minimally important differences as per the PROMIS scales, *u*—Unavailable, *N/A*—not applicable to Pompe disease (added to complete the list of GI related issues in the reference populations and to compare the characteristics between the two reference populations); * *p*-value was < 0.05 comparing GP versus GI clinical samples [28]. ** The most common were intestinal surgery (N = 72), symptomatic diverticular disease (N = 63), dyspepsia (N = 52), fecal incontinence (N = 44), pancreatitis (N = 25), celiac disease (N = 15), peptic ulcer (N = 15), and gastroparesis (N = 11). *** MIDs between baseline and follow-up T-scores were calculated to assess meaningful change in GI symptoms in patients with Pompe disease, over time. Note that patients within the GI-sample and LOPD groups were allowed to endorse more than one GI symptom during reporting (in our study and the other published articles). The published articles which describe the mean T-scores for the two control groups (GP and GI) [28] and the MIDs for longitudinal analyses [29] were last accessed on 3 June 2020. + is used to indicate an increase in the T-scores from baseline values, − is used to indicate a decrease in the T-scores from baseline values, and ± indicates either an increase or decrease from the baseline values.

## Data Availability

The data presented in this study are available on request from the corresponding author.

## Supplementary Materials

The following supplementary materials will be available online at https://www.mdpi.com/article/10.3390/jcm10153395/s1, Table S1: GI-focused medical history to assess the GI health of adult patients with Pompe disease, Table S2: Relationship between raw scores on PROMIS-GI symptom scales and clinical variables in patients with late-onset Pompe disease.
