# Supplementary material for: New Insights into Gastrointestinal Involvement in Late-Onset Pompe Disease: Lessons Learned from Bench and Bedside
Source: J Clin Med. 2021 Jul 30;10(15):3395. doi: 10.3390/jcm10153395 (PMC8347662; doi:10.3390/jcm10153395)
Supplement: Supplementary file 1 [file jcm-10-03395-s001.zip › jcm-1252921-supplementary marerials.pdf]

Supplementary Table S1. GI-focused medical history to assess the GI health of adult patients with Pompe disease.

1) Have you been diagnosed with a gastrointestinal (GI) disease or disorder by a physician? If yes, please list. (Examples include but are not limited to: swallowing dysfunction, esophageal dysmotility, GERD/acid reflux, gastritis, peptic ulcer disease, delayed gastric emptying, gallstones or other gallbladder/biliary disease, irritable bowel syndrome/IBS, celiac disease, Crohn's disease, ulcerative colitis, rectal sphincter dysfunction, fecal incontinence, etc.)
2) Have you been evaluated by a gastroenterologist (GI physician)?
3) Have you undergone an upper endoscopy? If so, for what reason?
4) Have you undergone a colonoscopy? If so, for what reason?
5) Have you been diagnosed with TMJ (temporomandibular joint) dysfunction?
6) Have you been told that you have tongue weakness by a medical professional (physician, therapist, etc.)? A history of difficulty with chewing, especially meats, any jaw fatigue? Any history of things getting stuck in throat? Difficulty with clearing food with tongue?
7) How many stools do you typically have per day?
8) If you do not have stools daily, how many stools do you typically have per week?
9) Are your GI symptoms worse at any time period of the day? Please state which symptom(s) and its association(s). If the question does not apply to you, please answer N/A.
10) Are your GI symptoms temporally associated with meals? Please state which symptoms(s) and its association(s). If the question does not apply to you, please answer N/A.
11) Do you take medications to relieve GI symptoms? If so, which medications and how often?
12) Do you consider your GI symptoms to be one of your top three symptoms that affects your quality of life?
13) From your recollection, did your GI symptoms start bothering you before or after your diagnosis of Pompe disease? Please state how long before/after to the best of your knowledge.
14) Has there been a change in your GI symptoms since you started treatment with ERT? Please state what changed and how it changed.
15) Is there worsening of GI symptoms after ERT (same day or up to 48 hours after)? Describe.
16) Do you have any other comments or concerns regarding GI issues?

Supplementary Table S2. Relationship between raw scores on PROMIS-GI symptom scales and clinical variables in patients with late-onset Pompe disease.

**Statistical relationships which were close to being significant or significant**		**p-value**
belly pain (raw score)	patient's age	0.0471*
diarrhea (raw score)	sex (male)	0.0686
swallowing (raw score)	CPK	0.0740
incontinence (raw score)	CPK	0.0619
swallowing (raw score)	FVC % predicted (supine) – inverse relationship	**0.0036****
diarrhea (raw score)	FVC % predicted (supine) – inverse relationship	0.0450*
incontinence (raw score)	FVC % predicted (supine) – inverse relationship	0.0264*

*p-value < 0.05, **p-value using Bonferroni correction <0.006.
